# A case report of novel mutation in *PRF1* gene, which causes familial autosomal recessive hemophagocytic lymphohistiocytosis

**DOI:** 10.1186/s12881-017-0404-9

**Published:** 2017-05-03

**Authors:** Mohammad Reza Bordbar, Farzaneh Modarresi, Mohammad Ali Farazi Fard, Hassan Dastsooz, Nader Shakib Azad, Mohammad Ali Faghihi

**Affiliations:** 1 Hematology Research Center, Shiraz University of Medical Sciences, Shiraz, Iran; 2 Center for Therapeutic Innovation, Department of Psychiatry and Behavioral Sciences, University of Miami Miller School of Medicine, 1501 NW 10th Ave, BRB 508, Miami, FL 33136 USA; 3 Comprehensive Medical Genetic Center, Shiraz University of Medical Sciences, Shiraz, Iran

**Keywords:** Hemophagocytic Lymphohistiocytosis (HLH), *PRF1*, Case report, Novel mutation

## Abstract

**Background:**

Hemophagocytic Lymphohistiocytosis (HLH) is a life-threatening immunodeficiency and multi-organ disease that affects people of all ages and ethnic groups. Common symptoms and signs of this disease are high fever, hepatosplenomegaly, and cytopenias. Familial form of HLH disease, which is an autosomal recessive hematological disorder is due to disease-causing mutations in several genes essential for NK and T-cell granule-mediated cytotoxic function. For an effective cytotoxic response from cytotoxic T lymphocyte or NK cell encountering an infected cell or tumor cell, different processes are required, including trafficking, docking, priming, membrane fusion, and entry of cytotoxic granules into the target cell leading to apoptosis. Therefore, genes involved in these steps play important roles in the pathogenesis of HLH disease which include *PRF1*, *UNC13D* (*MUNC13-4*), *STX11*, and *STXBP2* (*MUNC18-2*).

**Case presentation:**

Here, we report a novel missense mutation in an 8-year-old boy suffered from hepatosplenomegaly, hepatitis, epilepsy and pancytopenia. The patient was born to a first-cousin parents with no previous documented disease in his parents. To identify mutated gene in the proband, Whole Exome Sequencing (WES) utilizing next generation sequencing was used on an Illumina HiSeq 2000 platform on DNA sample from the patient. Results showed a novel deleterious homozygous missense mutation in *PRF1* gene (NM_001083116: exon3: c. 1120 T > G, p.W374G) in the patient and then using Sanger sequencing it was confirmed in the proband and his parents. Since his parents were heterozygous for the identified mutation, autosomal recessive pattern of inheritance was confirmed in the family.

**Conclusions:**

Our study identified a rare new pathogenic missense mutation in *PRF1* gene in patient with HLH disease and it is the first report of mutation in *PRF1* in Iranian patients with this disease.

## Background

Hemophagocytic lymphohistiocytosis (HLH) is an uncommon hyper-inflammatory syndrome with high mortality associated with different conditions, including neoplastic, infectious, autoimmune, or hereditary diseases [1]. Neuropathologic findings and neurologic symptoms have been identified approximately in 75% of pediatric cases, including seizures, meningitis, encephalopathy, ataxia, hemiplegia, cranial nerve palsies, mental status changes, or simply irritability [2]. In magnetic resonance imaging (MRI) studies some brain findings may be observed, which include the increase of nodular parenchymal lesions, leptomeningeal enhancement, demyelization, and atrophy [3]. The incidence of HLH has been estimated to be 1 to 225 per 300 000 live births and is reported in all ages, races and both genders [1, 2, 4]. HLH is grouped into two forms, which include familial type due to genetic mutations affecting the cytotoxic function of T lymphocytes and natural killer (NK) cells or acquired form presenting in different conditions such as infectious, malignant, rheumatologic, or metabolic diseases [1, 5]. HLH is a medical emergency and must be suspected in patients affected by unexplained cytopenias and fever at any age [6]. There are no any gold standard confirmatory laboratory tests for HLH, which makes it difficult to confirm the disorder in patients. This is due to the fact that laboratory testing can show false negative results and lack specificity, or may take times to perform the tests which are not useful in a clinical emergency [7].

The diagnostic criteria for acquired HLH include fever; cytopenias affecting at least 2 of 3 lineages in the peripheral blood; splenomegaly; hyperferritinemia; hemophagocytosis in the bone marrow, spleen, or lymph nodes; hypertriglyceridemia and/or hypofibrinogenemia; low or absent NK-cell activity determined by the 51-Cr release assay; and high levels of sCD25. Five of these eight criteria are essential for diagnosis of acquired HLH, but in familial cases with a known genetic abnormality (FHL with mutations), the diagnosis can be conducted without consideration of these five criteria [8–10]. Five different forms of FHL have been described based on defects in different genetic material and genes, including chromosome arm 9q mutations (FHL1), *PRF1* (FHL2), *UNC13D (MUNC13-4)* (FHL3), *STX11* (FHL4), and *STXBP2 (MUNC18-2)* (FHL5) [11–17]. To date, several mutations have been reported across exons and exon-intron boundaries of these genes. Therefore, mutation screening for HLH is complicated. However, molecular genetic approaches using next generation sequencing can be very useful in identification of familial cases suspected for HLH. Therefore, the purpose of this study was to identify disease causing mutation in a boy diagnosed with HLH.

## Case presentation

An 8-year-old boy was admitted to the hematology department in Namazi Hospital (Shiraz, Iran) due to clinical findings such as fever, jaundice, hepatosplenomegaly, and pancytopenia. Laboratory studies showed notable abnormal findings related to liver function tests and coagulation profile. He had an increased level of AST, ALT, LDH, total and direct bilirubin, prolonged prothrombin time (PT), and activated partial thromboplastin time (aPTT), serum ferritin (nearly 1000 ng/ml), and low level of fibrinogen, total protein, and albumin. A high titer of Ebstein-Barr virus (EBV) viral capsid antigen IgM antibody proved an acute EBV infection. Serological markers for hepatitis A, B, and C were negative, and antibody titers for autoimmune hepatitis were within normal range. Moreover, Wilson disease was ruled out by measuring serum ceruloplasmin and 24-h urine copper. Liver biopsy and bone marrow aspiration followed by biopsy was inconclusive with non-specific findings. Laboratory tests such as defective killing activity of either CD8 or NK cells, were not available in our center or elsewhere in Iran.

**Fig. 1:**
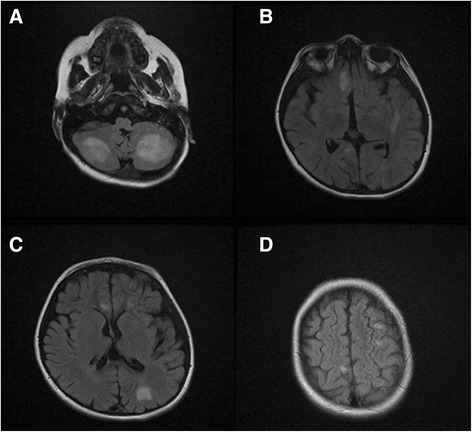
**a**, **b**, **c** and **d** Axial Flair sequences of brain MRI, which reveal numerous variable size and irregular shape hypersignal areas involving cerebral hemispheres, cerebellar hemispheres, pones and cerebral peduncles, mostly located in the corticomedullary junction and deep white matter in favor of HLH CNS involvement

The proband was product of consanguineous marriage (first-degree cousins) and there were no any documented HLH disease phenotype, immune disorders, hepatic diseases and blood malignancies in the immediate and extended family. The patient was suspected as a case of HLH according to HLH-2004 protocol [9] given the fact that he fulfilled the necessary criteria mentioned above. He was treated with dexamethasone, cyclopsporin and etoposide, but soon after starting treatment he showed dramatic responses with resolution of fever and correction of hepatitis, pancytopenia and bleeding tendency. Gradually, the patient developed clinical signs of the central nervous system (CNS) involvements such as convulsion, ataxia, spasticity and slurred speech. But cerebral spinal fluid (CSF) analysis for cell count, protein and cytology were normal. Brain MRI with and without contrast injection revealed spots of white matter hypersignal intensities on T2 and FLAIR images which were in favor of CNS involvement in HLH [18] (Fig. 1). Thus, we added intrathecal methotrexate and hydrocortisone to his treatment regimen, and searched for an HLA-matched donor for BM transplantation.

**Fig. 2:**
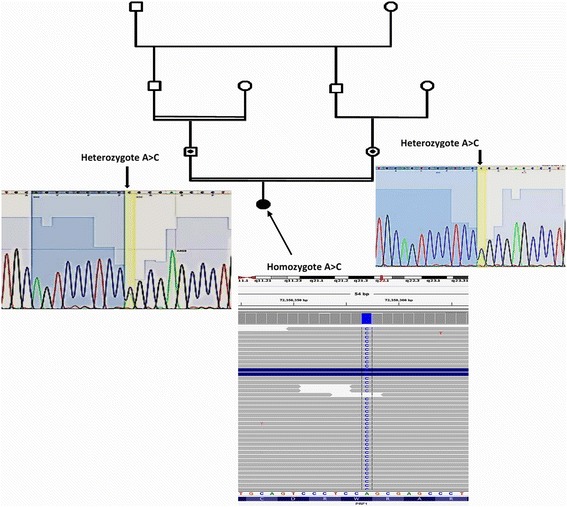
The proband is a boy with hepatitis and pancytopnea and his parents has consanguineous marriage. NGS results indicate homozygous mutation in *PRF1* gene in the proband as visualized using Integrative Genome Viewer (IGV) and using Sanger sequencing presence of the identified heterozygous mutation in *PRF1* gene was confirmed in the parents

**Fig. 3:**
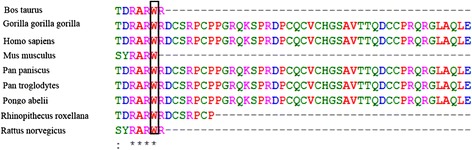
Comparative amino acids alignment of perforin protein across all Kingdoms. The W374 residue is highly conserved during evolution. The conserved tryptophan residue is shown in the *rectangular box*. Protein sequences were obtained from National Center for Biotechnology (NCBI). Symbols: (*)—identical amino acids; (:) — just similar amino acids

To determine whether the patient is a case of familial HLH, an unbiased next generation DNA sequencing which covered the entire coding exons was performed for detection of mutation in genes involved in FHL. We performed whole exome sequencing utilizing next generation sequencing on an Illumina platform on DNA sample from the patient. Detail of sample alignment is listed in Table 1. The text files of sequences were aligned using BWA aligner tool and variants were identified using GATK and annotated with the use of annovar software. In total, more than 120 K annotated variants were identified with hetero/homo ratio of 1.7, which then were filtered based on their frequency, location, functional consequences, inheritance pattern and more importantly clinical phenotype. Results revealed a novel homozygous missense mutation in *PRF1* gene (NM_001083116: exon3, c.1120 T > G, p.W374G, position 72,358,357 on chromosome 10). Homozygous mutations in *PRF1* gene, which is encoded for perforin 1, have been previously reported to cause type-2 of familial HLH, (OMIM number 603553) [19], having an autosomal recessive pattern of inheritance. Using Sanger sequencing, the new identified mutation was confirmed in the proband (as homozygote mutation) and his parents (as heterozygote mutation) (Fig. 2). This mutation has not previously been reported and this is the first report of mutation of *PRF1* gene in Iranian patients affected by HLH. Following evidences confirm that this mutation can lead to FHL2: 1-Whole exome sequencing only identified this mutation to be the main cause of FHL2 in the patient. 2- As can be seen in Fig. 2, Sanger sequencing confirmed the mutation in the proband and based on identified heterozygote mutation in his parents, the inheritance pattern must be an autosomal recessive. 3- Bioinformatics software such as polyphen, SIFT, LRT, Mutation Taster, FATHMM, Radial SVM and Mutation Assessor software are predicted that this variant will be damaging (Table 2) 4- As shown in Fig. 3, the comparative amino acids alignment of perforin 1 protein across all Kingdoms using multiple sequence alignment analysis using T-Coffee Multiple Sequence Alignment Program revealed that this amino acid is highly conserved during evolution. 5. In addition, a substitution from tryptophan amino acid with an aromatic side chain (at position 374) to glycine amino acid with a nonpolar and small hydrogen side chain can create major problem in the protein. Thus, this mutation in *PRF1* gene is extremely pathogenic in our patient with FHL2.

**Table 1:** Whole exome sequencing detail of coverage and number of reads

Type	Value	Type	Value
Number of mapped reads	41,674,840	Percent reads on target	95.70%
Percent assigned reads	95.70%	Average reads per amplicon	136
Uniformity of coverage	86.30%	Regions with at least 100 reads	53.69%
Regions with at least 1 read	99.54%	Regions with at least 500 reads	0.70%
Regions with at least 20 reads	90.02%	Regions reading end-to-end	35.97%
Regions with no strand bias	85.64%	Total aligned base reads	7,342,243,527
Bases in target regions	57,742,646	Total base reads on target	6,979,820,754
Percent base reads on target	0.95	Uniformity of base coverage	0.85
Average base coverage depth	121	Target bases with no strand bias	78.31%
Target base coverage at 1×	99.18%	Target base coverage at 100×	47.95%
Target base coverage at 20×	87.91%	Target base coverage at 500×	0.62%
Percent end-to-end reads	58.98%	mapping rate	99.10%
AQ17	92.21%	AQ20	87.51%

**Table 2:** Bioinformatic analysis of new identified mutation (p.W374G) in *PRF1* gene in the proband

Chr^a^	Start	Ref^b^	Alt^c^	Gene	Zygocity	Function	Freq^d^	dbSNP	
10	72358357	A	C	PRF1	Homo	Non-synonymous	0	0	
SIFT	Pred^e^	Polyphen	Pred	Mutation Taster	Pred	Mutation Assessor	Pred	FATHMM	Pred
0.1	T	0.958	Damaging	1	Damaging	2.71	Medium	-.2.83	Damaging
VEST3	CADD	Phred	GERP++_RS	Raddial SVM	Raddial SVM	LR_score	LR_ pred	Phylo P46way	Phylo P100_vert
0.965	3.221	16.79	5.83	0.452	Damaging	0.707	Damaging	2.212	1.696

^a^Chromosome

^b^Reference

^c^Alteration

^d^Frequency

^e^Prediction

## Discussion

*PRF1* which is located on the long arm of the chromosome 10 (10q22.1) is coded for perforin 1 that is functionally and structurally similar to complement component 9 (C9) involved in the complement system. Both proteins produce transmembrane tubules and able to lyse non-specifically various target cells. Perforin 1 is one of the essential cytolytic proteins of cytolytic granules, and it is well recognized to be a major effector molecule for T-cell- and natural killer-cell-mediated cytolysis. It has an important role in killing the "non-self" targets recognized by the immune system, for instance, in transplant rejection or some autoimmune diseases [20]. It plays a key role in secretory granule-dependent cell death, and in defense against virus-infected or neoplastic cells [12, 21, 22, 23]. Since the gene product is involved in killing virus-infected cells and it was impaired in our patient, his EBV infection might be due to the defect of this gene.

Mutations and variants in *PRF1* gene are also documented in other disorders which include perforin deficiency [24], multiple sclerosis [25], type 1 diabetes [26], Non-Hodgkin lymphoma, and leukemia [27, 28]. Up to now, more than 115 pathogenic gene variants have been reported in this gene, mainly missense and nonsense mutations. In general, severity of the disease depends on the residual activity of the perforin [29, 30].

*PRF1* mutations are responsible for approximately 20% of familial cases of HLH, with high frequencies in North America (approximately 50%), Japan (40%), and Turkey (30% [17, 31]. FHL2 is clinically characterized by fever, edema, hepatosplenomegaly, and liver dysfunction. In addition, neurologic impairment, seizures, and ataxia are frequent in this type [32]. It has been reported that laboratory studies can show pancytopenia, coagulation abnormalities, hypofibrinogenemia, and hypertriglyceridemia [33]. However, with advances in sequencing technologies, the disease is more readily detectable and secondary prevention for families become accessible. Although heterozygous carriers are not apparently sick, they are at risk of passing this deleterious mutation into their offspring. Therefore, *PRF1* genetic test must be requested for individuals with abnormalities suspected for HLH and should be offered to family members of known patients who intend to have consanguineous marriage. Since there is a high rate of monogenic disorders in Iran, particularly in rural areas where first-degree marriages are more common, finding and reporting rare novel pathogenic mutations would be extremely important for subsequent prevention of inherited disorders with homozygous pattern of inheritance.

Early molecular diagnosis and initiation of treatment for familial type-2 HLH is lifesaving [34]. In addition to the prevention of new cases of inherited disease in families with consanguineous marriages, identification of novel disease-causing mutations can provide proper molecular diagnosis of HLH, which will help to consider more reliable therapeutic approaches. While there is no cure for the HLH disease, recent advances regarding other monogenic disorders have provided several potential therapeutic options which include gene therapy, cell therapy, enzyme replacement therapy and bone marrow transplant. Symptomatic and supportive treatments for the hepatic and neurological abnormalities are the current available options, but do not significantly change the clinical course and outcome. Allogeneic hematopoietic stem cell transplantation is recommended and frequently used with promising outcomes [35]. Future therapies with the use of patient-derived iatrogenic pluripotent stem cells (iPSCs) [36], combined with CRISPR/CAS9 gene editing techniques [37] might help generate hematopoietic stem cell autograph transplantation.

However, with the limited available therapeutic option for most of the HLH patients, molecular diagnosis might enable geneticists and pediatricians to provide informative genetic counseling, perform prenatal diagnosis, and implement prevention measures for such patients. Therefore, genetic counseling should be recommended to all individuals with HLH disease and families for their next pregnancies and for other family members who want to have consanguineous marriages.

## Conclusions

In summary, a rare pathogenic mutation in *PRF1* gene was identified in our patient with FHL2 disorder, proving the link between *PRF1* gene mutations, hepatitis, neurologic manifestations, and pancytopenia in patients with HLH. Our study may help to establish an appropriate genetic counselling and prenatal diagnosis for individuals at the high risk of HLH disorder.

## Abbreviations

ALT: Alanine transaminase
APTT: Activated partial thromboplastin time
AST: Aspartate transaminase
CNS: Central nervous system
CSF: Cerebral spinal fluid
EBV: Epstein-Barr virus
FHL: Familial Hemophagocytic Lymphohistiocytosis
HLH: Hemophagocytic Lymphohistiocytosis
iPSCs: Iatrogenic pluripotent stem cells
LDH: Lactate dehydrogenase
MRI: Magnetic resonance imaging
*PRF1*: Perforin 1
PT: Prothrombin time
